# Posterior Polar Central Choroidal Dystrophy: A Case Report

**DOI:** 10.4274/tjo.56873

**Published:** 2017-10-27

**Authors:** Funda Dikkaya, Mustafa Özsütçü, Merve Özbek, Sevil Karaman Erdur, Fevzi Şentürk

**Affiliations:** 1 İstanbul Medipol University Faculty of Medicine, Department of Ophthalmology, İstanbul, Turkey

**Keywords:** Posterior polar central choroidal dystrophy, fluorescein angiography, fundus autofluorescence imaging, optical coherence tomography

## Abstract

A 52-year-old male presented with a 25-year history of decreasing vision. Best corrected visual acuity was 0.3 in his right and 0.2 in his left eye. Fundoscopic examination showed bilateral symmetric atrophy of the retinal pigment epithelium and choriocapillaris in the posterior polar areas between vascular arcades and surrounding the optic disc. On fluorescein angiography, the large choroidal vessels beneath these affected regions were easily seen. Fundus autofluorescence imaging showed clearly defined hypoautofluorescent areas that corresponded to the aforementioned lesions. Atrophy of the choriocapillaris and outer retinal layer were detected in optical coherence tomography. Photopic and scotopic responses were subnormal in flash electroretinogram (ERG), and responses were also minimal in pattern ERG and multifocal ERG. The patient was diagnosed with posterior polar central choroidal dystrophy. We aimed to present the results of fluorescein angiography, fundus autofluorescence imaging, optical coherence tomography, and electrophysiological tests in this rare case of posterior polar central choroidal dystrophy.

## INTRODUCTION

Posterior polar central choroidal dystrophy is a form of choroidal dystrophy characterized by loss of retinal pigment epithelium (RPE) and choriocapillaris. Involvement occurs in the posterior fundus within the vascular arcades and sometimes surrounding the optic nerve. This term was first used by Yannuzzi^1^ and since then there have been no further reports of this rare condition. In this case report, we present the results of fluorescein angiography, fundus autofluorescence (FAF) imaging, optical coherence tomography (OCT) and electrophysiological testing in this rare disease.

## CASE REPORT

**Figure 1:**
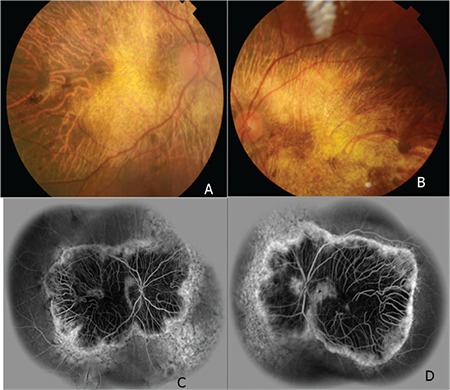
Right (A) and left (B) fundus images showing retinal pigment epithelium and choriocapillaris atrophy in the posterior polar areas between vascular arcades and around the optic nerve. Right (C) and left (D) fluorescein angiography demonstrating loss of choriocapillaris and retina pigment epithelium

**Figure 2:**
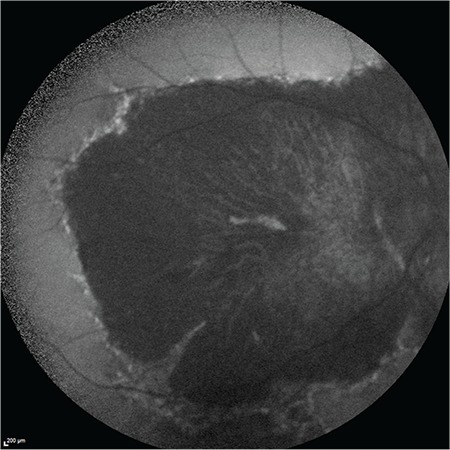
Fundus autofluorescence imaging of the right eye showing hypoautofluorescent areas corresponding to the atrophic area

**Figure 3:**
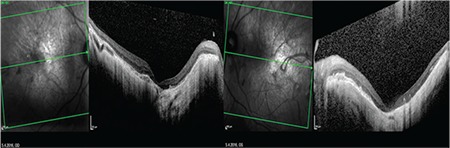
Optical coherence tomography images showing atrophy of the choriocapillaris and outer retinal layer

**Figure 4:**
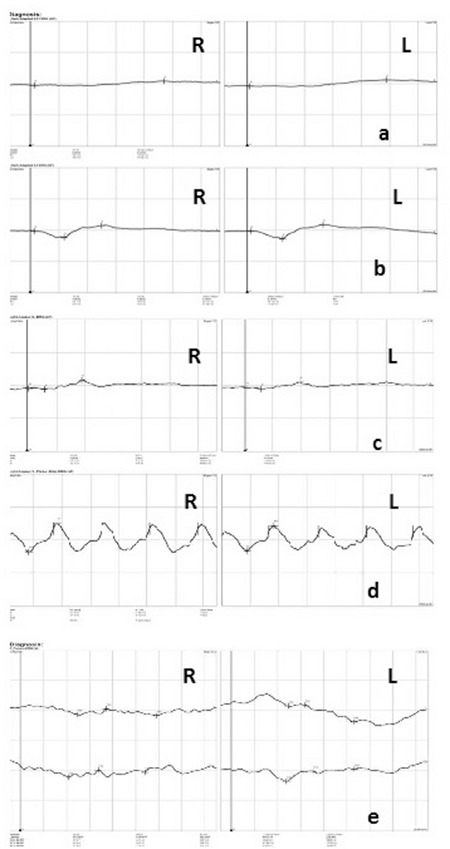
Full-field electroretinogram of right (R) and left (L) eyes. a) Dark adapted 0.01 ERG, b) Dark adapted 3.0 ERG, c) Light adapted 3.0 ERG, d) Light adapted 3.0 flicker 30 Hz ERG, e) Pattern ERG

**Figure 5:**
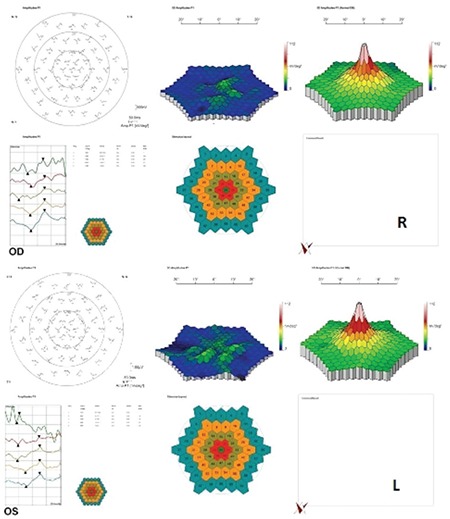
Multifocal electroretinogram of right (R) and left (L) eyes

**Figure 6:**
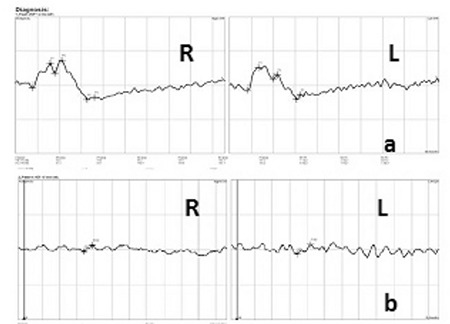
Visual evoked potential results of right (R) and left (L) eyes. a) Flash VEP, b) Pattern VEP

A 52-year-old male patient had a 25-year history of decreasing vision in both eyes. He denied any family history of similar ocular disorders. Best corrected visual acuity was 0.3 in his right and 0.2 in his left eye with Snellen testing. His refraction was -3.00 180°-0.75 in the right eye and -3.50 160°-0.50 in the left eye. Slit-lamp examination was normal in both eyes and intraocular pressure was 15 mmHg in the right eye, 16 mmHg in the left eye. Fundoscopic examination showed bilateral symmetrical RPE and choriocapillaris atrophy in the posterior polar areas between the vascular arcades and around the optic nerve. No flecks or drusen were observed (Figure 1A, B). On fluorescein angiography, large choroidal vessels were easily observed beneath these affected regions due to loss of the pigment epithelium and choriocapillaris (Figure 1C, D). FAF imaging showed clearly defined hypoautofluorescent areas that corresponded to the aforementioned lesions (Figure 2). Atrophy of the choriocapillaris and outer retinal layer were detected in OCT (Figure 3). Central macular thickness was about 58 μm and central choroidal thickness was about 118 μm for both eyes. Electroretinogram (ERG) and visual evoked potential (VEP) revealed that the patient’s b-wave amplitude on dark-adapted 0.01 ERG (rod response) was below the normal limit in both eyes. On dark-adapted 3.0 ERG, both a- and b-wave implicit times were prolonged in both eyes, and a-wave and b-wave amplitudes were reduced in both eyes. Light-adapted 3.0 ERG (cone response) revealed reduced a- and b-wave amplitudes. Pattern ERG responses were minimal. On light-adapted 30 Hz flicker ERG 30 Hz amplitudes were attenuated in both eyes. (16.2 µV, 13.5 µV) (normal range: 57-223 µV) (Figure 4, Table 1). There was a reduction in amplitude of all waveforms on the multifocal ERG (Figure 5). Pattern VEP responses were minimal and flash VEP was within normal limits (Figure 6).

## DISCUSSION

Primary choroidal dystrophies which affect the central macula are referred to as central areolar choroidal dystrophy, posterior polar central choroidal dystrophy, posterior polar annular dystrophy, posterior polar hemispheric dystrophy, and central and peripheral annular choroidal dystrophy.^1^ All forms of these choroidal dystrophies feature varying patterns of atrophy involving both the RPE and choriocapillaris. In posterior polar central choroidal dystrophy, the atrophic abnormality involves the posterior fundus within the vascular arcades and sometimes the area surrounding the optic nerve.^1^

OCT, which provides detailed analysis of retinal architecture, revealed atrophy of the choriocapillaris and outer retinal layers, and FAF images helped to localize the boundaries of the atrophic area.

The differential diagnosis of this condition included central areolar choroidal dystrophy, geographic atrophy, and pathologic myopia. We excluded pathologic myopia because the patient had refractive error less than -6.0 D. In central areolar choroidal dystrophy, atrophy of the RPE and choriocapillaris occurs in the foveal region and does not spread through the vascular arcade. The results of an electrophysiological study in a patient with central areolar choroidal dystrophy have been reported in the literature. Ponjavic et al.^2^ reported the result of full-field electroretinography in this disorder. They showed that the cone b-wave amplitude in ERG is decreased and the cone b-wave implicit time is prolonged. This result shows that although it is a choroidal disease, it also affects most or all of the retinal cones. Lotery et al.^3^ reported that pattern VEP and pattern ERG are the most sensitive electrophysiological tests which show abnormality in clinically normal but genetically affected central areolar choroidal dystrophy patients. Eventually in later disease stages, atrophy results in abnormal cone and rod responses on full-field ERG due to widespread photoreceptor dysfunction.

In another case report, full-field and multifocal ERG results of a patient with central areolar choroidal dystrophy showed normal photopic and scotopic responses in the full-field ERG and severely depressed retinal function of the perifoveal macula corresponding to the atrophic area in the multifocal ERG.^4^

Posterior polar central choroidal dystrophy affects a larger area than central areolar choroidal dystrophy and in our case the patient was in the advanced stage. Therefore, the patient demonstrated abnormal photopic and scotopic responses in full-field ERG as well as pattern VEP and pattern ERG. At the same time, all waves showed reduced amplitude in multifocal ERG.

Another disease in the differential diagnosis may be geographic atrophy, which is a devastating complication of age-related macular degeneration. Geographic atrophy of the RPE may be classified as drusen-related or neovascularization-related.^5^ Because there were no drusen or fibrovascular tissue in either eye and the findings were symmetrical, this diagnosis was excluded.

Based on all of these findings, we diagnosed the patient with posterior polar central choroidal dystrophy. We share the results of fluorescein angiography, FAF imaging, OCT, and electrophysiological tests in our case in order to remind clinicians of this rare disease.

## Figures and Tables

**Table 1:**
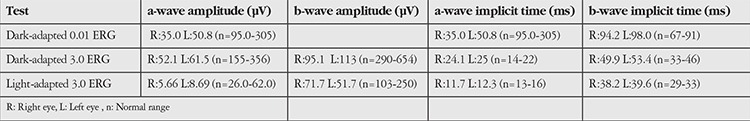
Results of full-field electroretinogram
